# Adeno-Associated virus 8 delivers an immunomodulatory peptide to mouse liver more efficiently than to rat liver

**DOI:** 10.1371/journal.pone.0283996

**Published:** 2023-04-11

**Authors:** Yuqing Wang, Ayrea Hurley, Marco De Giorgi, Mark R. Tanner, Rong-Chi Hu, Michael W. Pennington, William R. Lagor, Christine Beeton

**Affiliations:** 1 Department of Integrative Physiology, Baylor College of Medicine, Houston, Texas, United States of America; 2 Ambiopharm, Inc., North Augusta, South Carolina, United States of America; Fudan University, CHINA

## Abstract

Targeting the Kv1.3 potassium channel has proven effective in reducing obesity and the severity of animal models of autoimmune disease. Stichodactyla toxin (ShK), isolated from the sea anemone *Stichodactyla helianthus*, is a potent blocker of Kv1.3. Several of its analogs are some of the most potent and selective blockers of this channel. However, like most biologics, ShK and its analogs require injections for their delivery, and repeated injections reduce patient compliance during the treatment of chronic diseases. We hypothesized that inducing the expression of an ShK analog by hepatocytes would remove the requirement for frequent injections and lead to a sustained level of Kv1.3 blocker in the circulation. To this goal, we tested the ability of Adeno-Associated Virus (AAV)8 vectors to target hepatocytes for expressing the ShK analog, ShK-235 (AAV-ShK-235) in rodents. We designed AAV8 vectors expressing the target transgene, ShK-235, or Enhanced Green fluorescent protein (EGFP). Transduction of mouse livers led to the production of sufficient levels of functional ShK-235 in the serum from AAV-ShK-235 single-injected mice to block Kv1.3 channels. However, AAV-ShK-235 therapy was not effective in reducing high-fat diet-induced obesity in mice. In addition, injection of even high doses of AAV8-ShK-235 to rats resulted in a very low liver transduction efficiency and failed to reduce inflammation in a well-established rat model of delayed-type hypersensitivity. In conclusion, the AAV8-based delivery of ShK-235 was highly effective in inducing the secretion of functional Kv1.3-blocking peptide in mouse, but not rat, hepatocytes yet did not reduce obesity in mice fed a high-fat diet.

## Introduction

Adeno-associated virus (AAV) is a single-stranded DNA virus that can deliver genetic cargos that fit within the approximately 4.7 kb packaging limit [1]. Recombinant AAV (rAAV) is one of the safest gene delivery vehicles that can be packaged with a variety of naturally occurring and engineered capsid serotypes. Several clinical trials are currently using rAAV as a liver vector to treat monogenic diseases, such as spinal muscular atrophy, Duchenne muscular dystrophy, hemophilia A & B, Crigler-Najjar, or familial hypercholesterolemia [2–9]. While most liver-directed gene therapy efforts focused on correcting monogenic disorders, this technology has other important applications. A particularly promising approach involves using the liver as a biofactory for the production of therapeutic compounds [10, 11]. For example, AAV gene therapy has been used to produce enzymes deficient in lysosomal storage disorder diseases such as Pompe disease [12, 13], mucopolysaccharidosis type VI [14, 15], and Fabry disease [16]. However, most non-communicable chronic diseases currently treated with biologics require repeated injections over years or decades. Our goal is to use rAAV for the one-time delivery of a biologic for *in vivo* production to treat chronic diseases. As a biologic, we have chosen ShK-235, an analog of a sea anemone venom toxin modified to selectively block Kv1.3 potassium channels that can be produced recombinantly [17, 18].

The voltage-gated Kv1.3 potassium channel has gained interest as a therapeutic target for obesity, as mice lacking Kv1.3 or treated with a Kv1.3 blocker are protected from high-fat diet (HFD)-induced obesity [19, 20]. Kv1.3 is also a target for autoimmune diseases, as C-C chemokine 7 (CCR7)^-^ effector memory T (T_EM_) lymphocytes express high levels of Kv1.3 upon activation and blocking of Kv1.3 inhibits their function [21]. The small peptide ShK, produced by the sea anemone *Stichodactyla helianthus*, is one of the most potent blockers of Kv1.3 with an IC_50_ of 10 pM [22, 23]. However, ShK also blocks other Kv1.x channels and the KCa3.1 channel in the pM to low nM range [24]. ShK-186, known as Dalazatide, was generated by adding a pTyr to the N-terminus of ShK via an aminoethyloxyethyloxyacetyl (AEEA) linker [24, 25]. This modification maintained the pM affinity of the peptide to Kv1.3 and improved its selectivity for Kv1.3. ShK-186/Dalazatide reduces disease severity in rat models of delayed-type hypersensitivity (DTH), rheumatoid arthritis, and multiple sclerosis [26–30], and demonstrated an excellent safety profile after systemic administration in rodents, non-human primates, healthy volunteers, and patients with plaque psoriasis [26, 27, 30, 31]. However, ShK-186 must be synthesized as the AEEA linker prevents its recombinant expression. The recombinant production of biologics greatly reduces their cost of production when compared to chemical synthesis [32]. We, therefore, designed ShK-235 that lacks the pTyr-AEEA linker and can be expressed recombinantly. ShK-235 is highly potent in blocking Kv1.3 with an IC_50_ of 62 pM and 220-fold selectivity for Kv1.3 over other Kv and KCa3.1 channels, a selectivity similar to that of ShK-186 [17, 18]. ShK-186 has thus far been delivered via injection during all preclinical and clinical studies and the treatment of chronic conditions, such as obesity or autoimmunity requires repeated injections which can be associated with side effects ranging from injection site reactions to infections, leading to reduced patient compliance and thus decreased treatment efficacy [33].

To remove the need for frequent injections, we propose the sustained production of a selective Kv1.3 blocker by a patient’s own cells. To accomplish this goal, we designed AAV vectors based on serotype 8 (AAV8) to target the liver for expressing ShK-235 along with EGFP as a reporter. AAV8 is known to have a high tropism for murine liver [34, 35], which can be further restricted to this tissue with the use of hepatocyte-specific promoters [36]. We tested these vectors for hepatocyte transduction, ShK-235 production and secretion, and inhibitory blocking of the Kv1.3 channel in both mice and rats. We also determined if this hepatocyte-secreted ShK-235 could reduce weight gain in animals fed an HFD and inflammation in a model of DTH.

## Materials and methods

### Plasmid design and cloning

The control AAV plasmid expressing EGFP driven by the cytomegalovirus (CMV) enhancer element and the chicken β-actin promoter was previously obtained from Dr. Wilson and is publicly available (Addgene # 105530). ShK-235-expressing AAV transgene plasmids were constructed by standard molecular techniques using gene synthesis from IDT as the source of new sequences.

Expression of ShK-235 was driven by the cytomegalovirus (CMV) enhancer/beta-actin (CB) promoter combination. In the first two vectors designated GFP and SHK1, the complete Chicken β-actin promoter was used. For the smaller P2A containing constructs SHK2 and SHK3 we used a smaller version of the Chicken β-actin promoter, designated as CB*, which contains an internal deletion of the 34 bp sequence ggggggggcgcgcgccaggcggggcggggcgggg. Complete vector sequences are provided in S1 and S2 Figs.

In every vector, we fused the leader sequence from human Apolipoprotein A-I to the N-terminus of ShK-235 to drive expression and secretion from the liver. ApoA-I is the main protein constituent of high-density lipoprotein particles and is highly expressed by the liver and intestine. The ApoA-1 leader sequence encodes the signal peptide of human pre-pro-ApoA1 (MKAAVLTLAVLFLTGSQA) followed by six amino acids of the pro-ApoA1 sequence (RHFWQQ) which is subsequently cleaved during or immediately following secretion in the circulation by PCPE2/BMP-1 [37] (S3 Fig). The ShK-235 sequence encodes the immunomodulatory peptide: RSCIDTIPKSRCTAFKCKHSIKYRLSFCRKTCGTCA.

Internal ribosome entry site (IRES) elements have the advantage of providing bicistronic expression from a single mRNA without modification of native protein sequences. Ribosomal skipping peptides such as P2A generally drive higher expression of the downstream protein but result in the modification of both proteins with additional amino acids, which can potentially affect function.

The hybrid liver-specific promoter (HLP) sequence is identical to that reported by McIntosh J. et al. [36]. A diagram of all the AAV vectors used in this study is shown in S1 Fig. All transgene cassettes were fully verified by Sanger sequencing, and complete plasmid sequences are provided as S2 Fig and the sequence of the signal peptide and pro-region of Apo A1 used in our constructs is provided as S3 Fig.

All constructs contain both ShK-235 and EGFP. EGFP was included for ease of detection in tissue culture and in solid organs as ShK-235 is not immunogenic [18] and no antibodies exist against this peptide, requiring patch-clamp electrophysiology for its detection in conditioned media and body fluids.

### Transfection of HEK293T cells and detection of EGFP expression

HEK293T cells (ATCC, CRL-3216) were used to test the transgenes because of their high transfection efficiency (85–90%) compared to liver cancer cells whose transfection efficacy is <40%. HEK293T cells were cultured in Dulbecco’s Modified Eagle Medium (DMEM, Fisher Scientific) supplemented with 10% heat-inactivated fetal bovine serum (FBS) (Millipore Sigma) and 1% Penicillin/Streptomycin (VWR) at 37⁰C and 5% CO_2_. One million cells were plated in 100 mm dishes and used for transfection when at 60–70% confluency. Cells were transfected using polyethylenimine (PEI) and 20 μg of the specific plasmid were mixed with 60 μg of 1 mg/mL PEI solution in 1 mL of Opti-MEM media (ThermoFisher). The transfection mixture was added to the cells, incubated for 5 hours, and then replaced with fresh medium. ShK is a highly efficient folding peptide, even when making substituted analogs, and yields of the synthetic products range from 8 to 22% [38, 39]. When expressed in a eukaryote expression system, peptides with disulfide bonds are fully folded in the rough endoplasmic reticulum, requiring no additional steps to obtain a fully functional peptide [40]. EGFP fluorescence was imaged 72 hours post-transfection using a Leica DMIL LED Inverted Fluorescent Microscope with a 10x objective and 30 ms exposure times.

### AAV8 production and purification

AAV8 vectors were chosen because they have a very high tropism to the mouse liver [34, 35]. Expression can be further restricted to the liver using hepatocyte-specific promoters such as the hybrid liver-specific promoter (HLP) [36]. As an example, AAV8-HLP-Cre has been demonstrated to mediate very efficient Cre recombination in the liver, but not in any of the other 10 non-hepatic tissues examined [41]. Four AAV8 vectors were produced, as shown in Fig 2A, of which 2 vectors having the hybrid liver-specific promotor (HLP) to confine transgene expression to hepatocytes [36]. Several constructs were tested as we changed the promoter from CB to HLP, and the target cells from HEK293 to hepatocytes, which could have changed the expression levels of EGFP or ShK-235.

AAV8 vectors were generated as described [42, 43]. Briefly, the Adenoviral helper plasmid pAdDeltaF6 and the Rep/Cap packaging plasmid pAAV2/8 were obtained from the University of Pennsylvania Vector Core, and are now available on Addgene (#112867, #112864). The AAV-ShK transgene plasmids, which harbor the ITR’s from AAV2, were transfected along with pAdDeltaF6 and pAAV2/8 into HEK293T cells in 15 cm dishes using PEI. AAV vectors were harvested from cell pellets and purified using a single CsCl density gradient. Fractions containing AAV particles were identified based on refractive index and pooled, followed by dialysis in 100,000 molecular weight cutoff cassettes to remove CsCl. Purified AAV were concentrated in Amicon 100 kDa MWCO centrifugal concentration devices to a volume of 150 μl or less. AAV titers were measured following DNase digestion to remove unencapsidated DNA. The DNase-resistant vector genomes were determined in genome copies (GC) by qPCR relative to a standard curve of plasmid, using primers specific to the EGFP transgene. The yields of all constructs ranged from 1 E12 to 6 E12 GC per prep from 20 x 15 cm plates of HEK293T cells, comparable to other AAV8 vectors produced in the lab. Following titer determination, AAV were aliquoted and stored at -80°C until ready for use.

### Synthesis of ShK-235

ShK-235 was synthesized on a CEM Liberty Blue peptide synthesizer using an Fmoc-tBu strategy, as described [17, 18]. The peptide was synthesized starting with Fmoc-Ala-Wang resin. All couplings were mediated with diisopropyl carbodiimide and Oxyma. Following solid-phase assembly of the linear peptide chain, the peptide was cleaved from the solid support and simultaneously deprotected using Reagent K for 2 h at room temperature. The crude peptide was precipitated into ice-cold diethyl ether and washed thoroughly to remove cationic scavengers from the cleavage cocktail, dissolved in 50% aqueous acetic acid, then diluted in water and the pH adjusted to 8.0 with NH_4_OH. Disulfide bond formation was facilitated with reduced and oxidized glutathione according to previously used protocols for ShK [44]. The progress of folding was followed by reversed phase high-performance liquid chromatography (RP-HPLC) using a Phenomenex Luna C18 column using a gradient of acetonitrile versus H_2_O containing 0.05% trifluoroacetic acid (TFA) from 10–70% over 35 min. Folding of the three disulfide bonds was also confirmed by the loss of 6 mass units from the crude material as determined by electrospray ionization mass spectrometry (ESI-MS). The cyclized peptide was purified by preparative RP-HPLC using a linear gradient of 0.05% TFA in H_2_O versus 0.05% TFA in acetonitrile. Fractions with a purity >95% were pooled together and subsequently lyophilized. The lyophilized peptide was analyzed for purity by analytical HPLC and ESI-MS.

### Patch-clamp electrophysiology

Mouse L929 fibroblasts stably expressing mKv1.3 channels [45] were a kind gift from Dr. Chandy (Nanyang Technological University, Singapore). They were maintained in Dulbecco’s modified Eagle’s medium (DMEM) supplemented with 10% heat-inactivated fetal bovine serum (FBS), 4 mM L-glutamine, 1 mM sodium pyruvate, and 500 μg/mL G418 (Calbiochem/EMD Millipore, Billerica, MA) and cultured to 60~70% confluence.

Patch clamp assays were conducted at room temperature (20–25°C) in the whole-cell configuration using a Port-a-Patch system (Nanion Technologies, Livingston, NJ) connected to a HEKA EPC10-USB amplifier controlled with the PatchMaster software (HEKA, Germany) [17, 46–48]. The external solution contained 1 mM MgCl_2_, 160 mM NaCl, 4.5 mM KCl, 2 mM CaCl_2_, and 10 mM Hepes; pH was adjusted to 7.4 with NaOH. The internal solution contained 2 mM MgCl_2_, 145 mM KF, 10 mM Hepes, and 10 mM EGTA; The pH of the internal solution was adjusted to 7.4 with KOH. The osmolarity of both internal and external solution were adjusted to 290–320 mOsm. The holding potential was set to −80 mV, and the resistance of chips was 2–3.5 MΩ. Kv1.3 currents were elicited by 200 ms pulses from −80 to 40 mV applied every 30 s. Series resistance compensation of 80% was used when the current exceeded 2 nA.

The stock of synthetic ShK-235, the supernatants of transfected HEK293T cells, and the rodent serum samples were diluted in the external solution immediately before use and tested in triplicates without any further processing. Peak Kv1.3 currents were recorded before addition of the samples and after reaching steady-state block and results were analyzed using IGOR Pro software (WaveMetrics, Portland, OR) and the IC_50_ of channel block was calculated by nonlinear regression using Prism software (GraphPad Software, San Diego, CA).

### Detection of the 2A peptide and EGFP by Western blotting

Supernatants were collected from HEK293T cells transfected with the ShK-235 or EGFP plasmids. The protein level in supernatants was measured using the BCA assay (Thermo-Pierce #23227) and equal amount of proteins (50 μg) were loaded and separated by SDS-PAGE using 10% Bis-Tris gels (ThermoFisher, San Francisco, CA). Then, proteins were transferred onto nitrocellulose membranes (GE healthcare, Chicago, Illinois) followed by blocking with 4% blotto non-fat dry milk (ChemCruz, sc-2325, Dallas, TX) in PBS for 1 hour at room temperature. Blots were incubated with primary polyclonal rabbit anti-2A antibody diluted in blocking solution at room temperature for 3 hours (1:5000 dilution, ABS31, Millipore Sigma, St. Louis, MO), followed by secondary goat anti-Rabbit IRDye 800CW (1:10000 dilution, 926–32211, LI-COR, Lincoln, NE) antibodies at room temperature for 1 hour. Visualization was performed with Chemidoc (Bio-Rad, Hercules, CA). To detect EGFP, blots were incubated with primary rabbit anti-GFP antibody (1:3000 dilution, A-11122, Fisher-Scientific, San Francisco, CA) and mouse anti-beta Tubulin [E7] (1:500 dilution, hybridoma bank, IA) diluted in blocking solution overnight at 4 degrees, followed by secondary goat anti-rabbit 680 and anti-mouse 800 nm at 1:15000 incubated at room temperature for 1 hour. Visualization was performed with Odyssey and Image Studio, Intensity 5, 169 μm resolution.

### Animals

All experiments involving animals were performed under protocols approved by the Animal Care and Use Committee at Baylor College of Medicine. Inbred C57BL/6J mice, 5 weeks old, were purchased from the Jackson Laboratories (Mount Desert Island, ME) and inbred LEW/SsNHsd Lewis rats, 8–10 weeks old, from Envigo (Indianapolis, IN). C57BL/6J mice were chosen because this is the strain used in a prior study showing the efficacy of a Kv1.3 blocker in a model of high-fat diet-induced obesity [19]. Lewis rats were chosen for the active DTH study because this is the strain in which Kv1.3 blockers were found effective [49–51]. Sample sizes were determined using an online sample size calculator (heets://sample-size.net). Based on previous studies, we performed sample size calculations with α = 0.05, β = 0.2, and effect size of 3 g, as reported. For the obesity study, we also accounted for the possible loss of animals during the long-term treatment and decided on 9 mice per group.

All animals were housed in groups of 3 to enhance their well-being, with food and water *ad libitum* within a facility accredited by the Association for Assessment and Accreditation of Laboratory Animal Care. Research was performed in accordance with relevant guidelines and regulations and data are reported in accordance with ARRIVE guidelines. Both females and males were used and animals were randomly assigned to treatment groups.

Animals received a single injection of AAV, either intraperitoneally or intravenously, as indicated in the figure legends. Daily monitoring of animal behavior was conducted to assess any signs of pain or discomfort: immobility, huddled posture, inability to access food or water, ruffled fur, self-mutilation, hypothermia, dyspnea, >20% weight loss, or vocalization. No such indications were noted. At the end of each trial, blood was collected and animals were euthanized via exsanguination by cardiac puncture while under a surgical plane of anesthesia with isoflurane, as described [52].

### Obesity induction and monitoring

Mice were switched to HFD TD. 88137 (Envigo Teklad Diets, Madison, WI). On the same day, they were separated into four groups and received either a single intraperitoneal injection of 2E+11 genome copies (GC) of AAV8-GFP or AAV8-HLP1, or they received ShK-235 (0.5 mg/kg body weight, subcutaneously) or vehicle (P6N buffer; 10 mM sodium phosphate, 0.8% NaCl, 0.05% polysorbate 20, pH 6.0) injections every other day for the duration of the trials [30]. Each injection volume was 0.1 ml. Mice were weighed weekly, as was the food remaining in the cages and the food added to each cage.

### Active delayed-type hypersensitivity

Two weeks after the injection of AAV8, an active DTH was induced and monitored in Lewis rats, as described [53–55]. Rats were immunized subcutaneously in the flanks with 200 μl of a 1:1 emulsion of ovalbumin (Sigma, St. Louis, MO) and complete Freund’s adjuvant (Difco/Becton Dickinson, Franklin Lakes, NJ). One week later, rats were challenged with ovalbumin dissolved in saline in the pinna of one ear and a control injection of saline in the other ear under isoflurane anesthesia. At 24 hours post-challenge, ear inflammation was determined by measuring and comparing the ear thickness of the ovalbumin-challenged the saline-challenged ear from each rat using a spring-loaded micrometer (Mitutoyo, Japan). At the end of the experiment, rats were humanely euthanized.

### Immunohistochemistry

Tissues were fixed in 10% buffered formalin, embedded in paraffin, and sectioned by the Pathology & Histology Core at Baylor College of Medicine. Sections were deparaffinized with xylenes and hydrated through a gradient of ethanol before antigen retrieval using pre-heated sodium citrate buffer (10 mM, pH 6.5). Sections were incubated overnight in PBS + 5% goat serum + 5% BSA to block non-specific protein binding, followed by blocking of endogenous peroxidase and alkaline phosphatase using BLOXALL (Vector Laboratories, San Francisco, CA). Sections were washed and incubated in primary antibodies against GFP (Invitrogen, catalog # A11122, San Francisco, CA). For detection, we used ImmPRESS anti-rabbit IgG conjugated to peroxidase followed by ImmPACT DAB (Vector Laboratories, San Francisco, CA). Sections were then washed and dehydrated in an ascending ethanol gradient, immersed with xylenes, and mounted with Permount. Slides were imaged at 200x magnification on a Nikon Ci-L bright-field microscope in the Integrated Microscopy Core at Baylor College of Medicine. GFP expression in the liver slides was evaluated using IHC scores by the following criteria: *Score 1*: <10% positive, *Score 2*: 10–20% positive, S*core 3*: 25–50% positive, *Score 4*: 60–75% positive, *Score 5*: >80% positive. Five random fields were examined, and the average score data was used.

### Statistical analysis

All data are presented as mean ± SEM. Statistical analyses involving time courses or multiple groups were performed with a two-way ANOVA followed by Tukey’s post-test for multiple comparisons. Two-tailed Student’s t-tests were used for comparisons involving two groups. A *p* value <0.05 was considered to be significant. GraphPad Prism 5.0 (GraphPad Prism Inc., San Diego, CA) was used for statistical analyses.

## Results

### Design and *in vitro* validation of AAV-ShK-235 constructs

**Fig 1:**
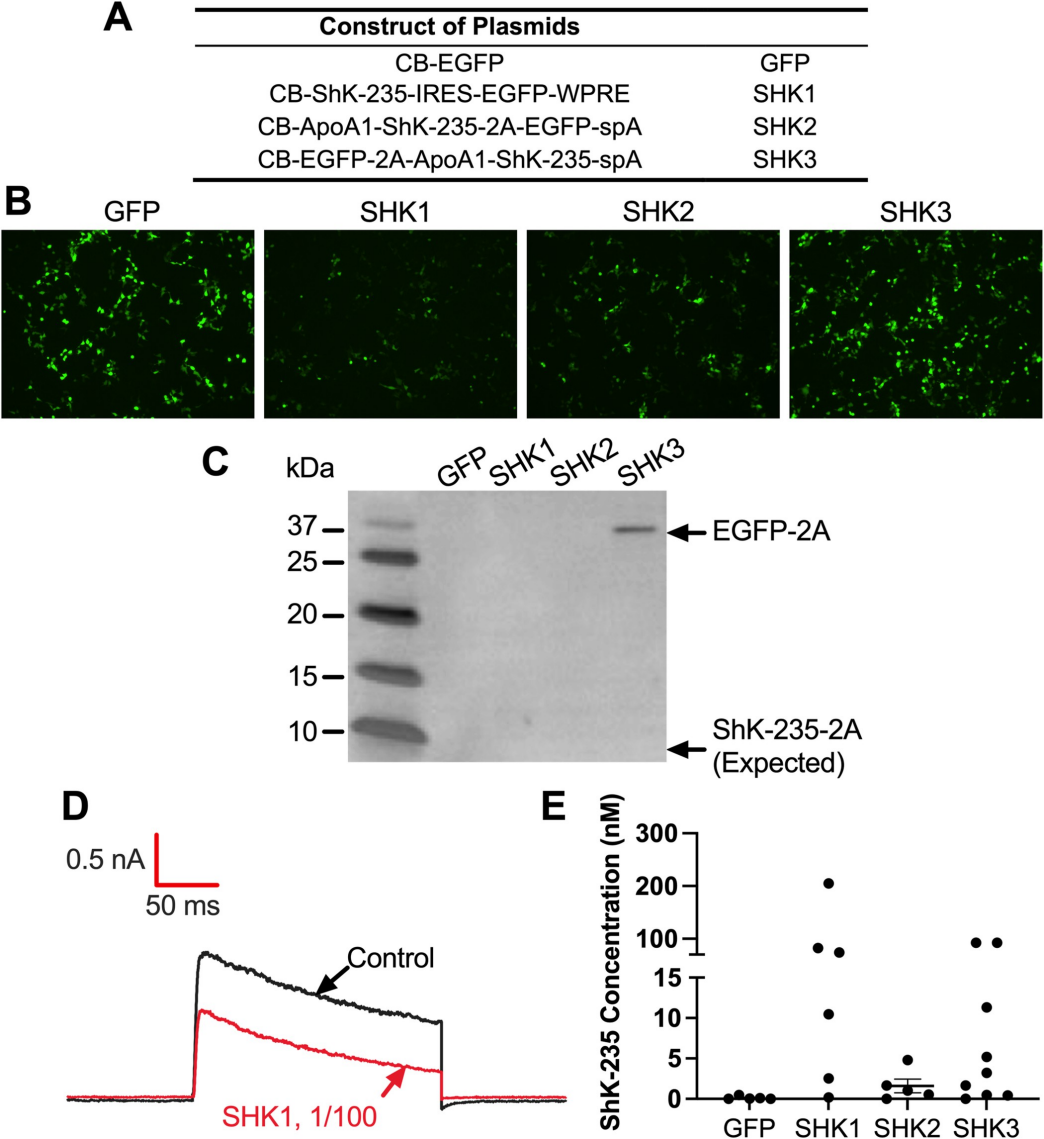
Design and characterization of AAV8-ShK-235 vectors. **(A)** Constructs used to express EGFP (GFP) or ShK-235 (SHK1, SHK2, and SHK3). **(B)** Representative fluorescence microscope images of HEK293T cells 72 hours after transfection with GFP, SHK1, SHK2, or SHK3 plasmids (20 μg of specific plasmid + 60 μg of 1 m/mL of PEI). **(C)** Western blot showing the detection of the 2A peptide in the supernatants of the HEK293T cells transfected with GFP control, SHK1, SHK2, or SHK3 plasmids, attached to either EGFP (32.7 kDa) or ShK-235 (4.1 kDa). Supernatants were tested without further purification or concentration. **(D)** Representative whole-cell Kv1.3 currents in L929 fibroblasts stably expressing mKv1.3 before (control) and after application of supernatants from HEK293T cells transfected with GFP control or SHK1, diluted 1:100. **(E)** ShK-235 concentration in supernatants from HEK293T cells transfected with GFP control, SHK1, SHK2, or SHK3 plasmids. Each data point represents a different recording. The horizontal bar represents the mean concentration.

To test the ability of the constructs (Fig 1A) to express EGFP *in vitro*, HEK293T cells were transfected and assessed by fluorescence microscopy (Fig 1B). The greatest EGFP fluorescence intensity was detected in SHK3 and the CB-EGFP control plasmid. The weakest EGFP expression was seen in SHK1, which was expected for a protein expressed from an IRES element (Fig 1B). SHK2 has a 2A peptide attached to the C-terminus of ShK-235, whereas SHK3 has the same peptide at the C-terminus of EGFP. To determine whether this affected either the secretion of ShK-235 produced by SHK2, Western blots were performed to detect the 2A peptide (Fig 1C and S6 Fig). As expected, the 2A peptide was not detected in the supernatants of the HEK293T cells transfected with EGFP or SHK1 as neither construct contains the 2A peptide. EGFP-2A was readily detectable in the supernatants of HEK293T cells transfected with SHK3 but no band was detected at the calculated molecular weight for ShK-235-2A in the supernatants of HEK293T cells transfected with SHK2. ShK-235-2A was also not detected in lysates of cells transfected with SHK2 (data not shown), suggesting a low production of this modified peptide, below the detection limit of Western blot and thus requiring the use of patch-clamp for detection. Patch-clamp is a functional assay and can therefore detect functional ion channel modulators at concentrations around their IC_50_. This technique was thus chosen to assess ShK-235 secretion by the transfected HEK293T cells. Known concentrations of synthetic ShK-235 were first tested to draw a dose-response curve of the block of Kv1.3 (S4 Fig), and used the curve to calculate the concentration of ShK-235 in each sample (Fig 1D and 1E). Except for the CB-EGFP control, all constructs led to detectable levels of ShK-235. SHK2 was the least efficient (mean concentration 1.6 nM, maximum 4.8 nM), while SHK1 (mean concentration 62 nM, maximum 205 nM) and SHK3 (mean concentration 23 nM, maximum 93 nM) induced the production and secretion of high levels of the functional peptide. All constructs containing ShK-235 induced the secretion of the peptide at concentrations well above its IC_50_ for Kv1.3 block of 70.9 ± 21.9 pM (S4A and S4B Fig).

### AAV8 delivery of GFP and ShK-235 to mouse liver

**Fig 2:**
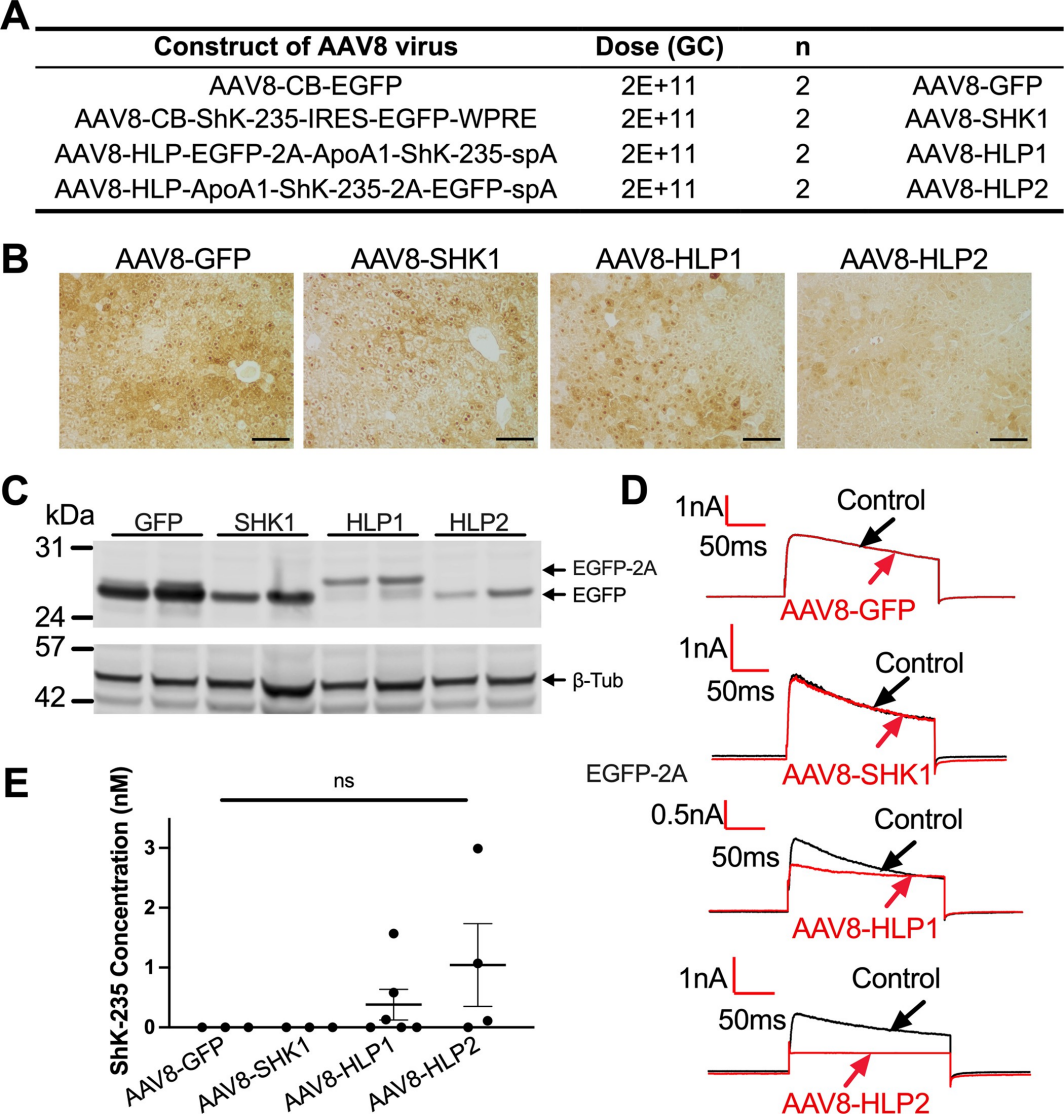
Efficient and specific GFP and ShK-235 peptide expression in mice liver following AAV8-based delivery. **(A)** AAV8 constructs and doses injected intraperitoneally into male mice. GC = genome copies. N = 8, or 2 male mice per group. **(B)** Representative IHC images of mouse livers showing EGFP expression. Scale bars = 100 μm. **(C)** Western blot analysis of EGFP in liver lysates from mice injected with AAV8 constructs in **(A)**, with β-tubulin (β-Tub) used as a loading control. **(D)** Representative whole-cell Kv1.3 currents in L929 fibroblasts stably expressing mKv1.3 before (control) and after application of serum from mice injected with AAV8-GFP, AAV8-SHK1, AAV8-HLP1, or AAV8-HLP2, diluted 1:50. Serum was collected 2 weeks post vector delivery. **(E)** ShK-235 concentration in serum of mice injected with AAV8-GFP, AAV8-SHK1, AAV8-HLP1, or AAV8-HLP2. Each serum sample was tested on 3 cells each.

Male mice received a single intraperitoneal injection of 2E+11 GC of the specific AAV8 vectors expressing only EGFP (AAV8-GFP), or both ShK-235 and EGFP (Fig 2A). AAV8-SHK1 had ShK-235 and EGFP separated by an IRES whereas AAV8-HLP1 and AAV8-HLP2 used the 2A peptide that, upon cleavage, was expected to remain attached to either EGFP (AAV8-HLP1) or to ShK-235 (AAV8-HLP2). Two weeks post-vector administration, the mice were euthanized and their serum and organs were collected for subsequent analyses. GFP was detectable by immunohistochemistry and Western blot in all livers (Fig 2B and 2C). A very low expression of EGFP was detected in the pancreas of animals injected with the AAV8-GFP control vector. In contrast, the ShK-235 expressing vectors did not show detectable expression in peripheral tissues (S5 Fig).

To determine if functional ShK-235 was produced *in vivo*, we tested if serum samples collected from the mice could block the Kv1.3 channel by whole-cell patch-clamp (Fig 2D and 2E). The sera from mice that received AAV8-GFP or AAV8-SHK1 had no detectable Kv1.3 block. In contrast, the serum of mice that received either AAV8-HLP1 or AAV8-HLP2 inhibited Kv1.3, and the calculated mean circulating ShK-235 concentration in mice injected with AAV8-HLP1 or AAV8-HLP2 was 0.38 nM or 1.04 nM, respectively (Fig 2E). These results suggest that a single intraperitoneal injection of the AAV8 vectors with the hepatocyte-specific promoter in mice led to liver-specific transduction and secretion of functional ShK-235 peptide in the short-term, the concentrations of which are well above the IC_50_ of ShK-235 for Kv1.3.

### AAV8-ShK-235 failed to protect mice from HFD-induced weight gain

**Fig 3:**
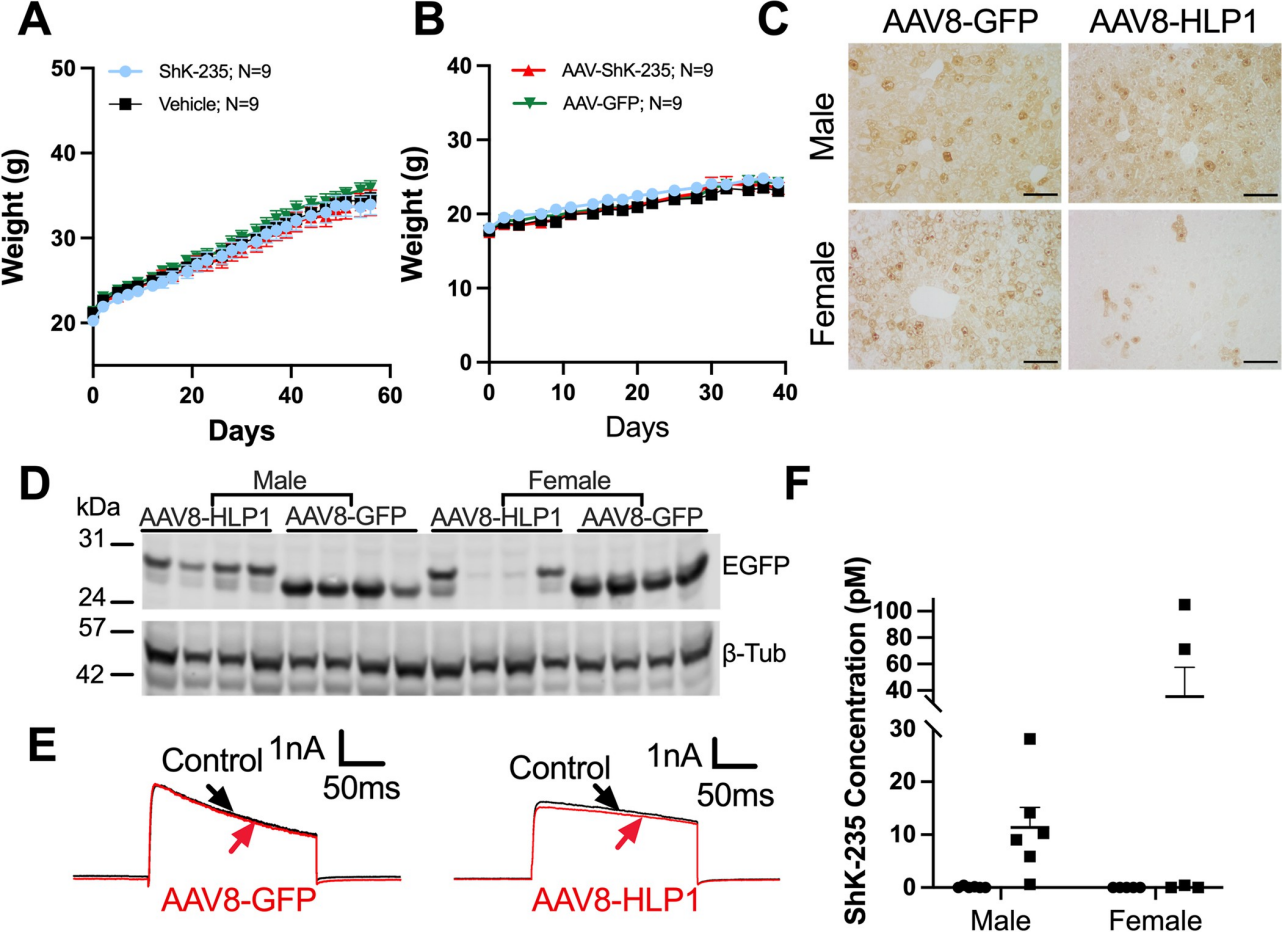
Lack of long-term secretion of ShK-235 by mouse hepatocytes. **(A, B)** Weekly weights of male **(A)** and female **(B)** mice after a single intraperitoneal injection of 2E+11 GC of AAV8-HLP1 (▲) or 2E+11 GC of AAV8-GFP (▼), or subcutaneous injection of 500 μg/kg synthetic ShK-235 (●) or of vehicle (■) every other day. N = 9 males and 9 females per group. **(C)** Representative IHC images of mouse livers showing EGFP expression. Scale bars, 100 μm. **(D)** Western blot analysis of male and female mouse liver lysates from obesity mice that received AAV8-GFP or AAV8-HLP1 injections, with β-tubulin used as a loading control. **(E)** Representative whole-cell Kv1.3 currents in L929 fibroblasts stably expressing mKv1.3 before (control) and after application of serum from mice injected with AAV8-GFP or AAV8-HLP1, diluted 1:50. **(F)** ShK-235 concentration in serum from mice injected with AAV8-GFP (●) or AAV8-HLP1 (■). N = 5–6 cells per group.

Knockout of Kv1.3 in mice and treatment with the Kv1.3 blocker ShK-186 prevent weight gain in C57BL/6J mice fed a HFD [19, 20]. ShK-235 is a recombinant analog of ShK-186 with similar potency and selectivity against Kv1.3, we therefore tested the efficacy of the AAV8-based delivery of ShK-235 in the high-fat diet-induced obesity model. As it takes weeks for mice to develop obesity, we also investigated if a single dose of AAV8 treatment can lead to long-term transgene expression of the functional ShK-235 peptide. On the day of switching from chow diet to HFD, four groups of mice received either a single intraperitoneal injection of AAV8-HLP1, AAV8-GFP virus, or a subcutaneous injection of ShK-235 or vehicle every other day. ShK-235 injection was used as a positive control. Eight weeks into our trial, none of the male mice showed differences in weight gain (Fig 3A) or food intake (S7A Fig). Upadhyay *et al*. reported a reduction in weight gain in male mice as early as 15 days after the start of treatment with ShK-186 [19]. This study was therefore terminated after 8 weeks for the males (Fig 3A) and after 5 weeks for the females (Fig 3B) and serum and organs of the mice were harvested for further assays. As with the males, no change in food intake was seen in females (S7A Fig). Livers from males expressed high levels of GFP 8 weeks after receiving AAV8-GFP or AAV8-HLP1 (Fig 3C and 3D). In contrast, the livers of the females still expressed high levels of GFP 5 weeks after receiving AAV8-GFP, but markedly less with AAV8-HLP1 (Fig 3C and 3D). We further determined that the concentration of ShK-235 in the serum of mice having received AAV8-HLP1 was only 0.6–28 pM in the males and 0–104 pM in the females (Fig 3E and 3F); well below the IC_50_ of Kv1.3 block by ShK-235. These results suggest lack of long-term secretion of ShK-235 by both male and female mouse hepatocytes.

### AAV8- ShK-235 failed to protect rats from a DTH

**Fig 4:**
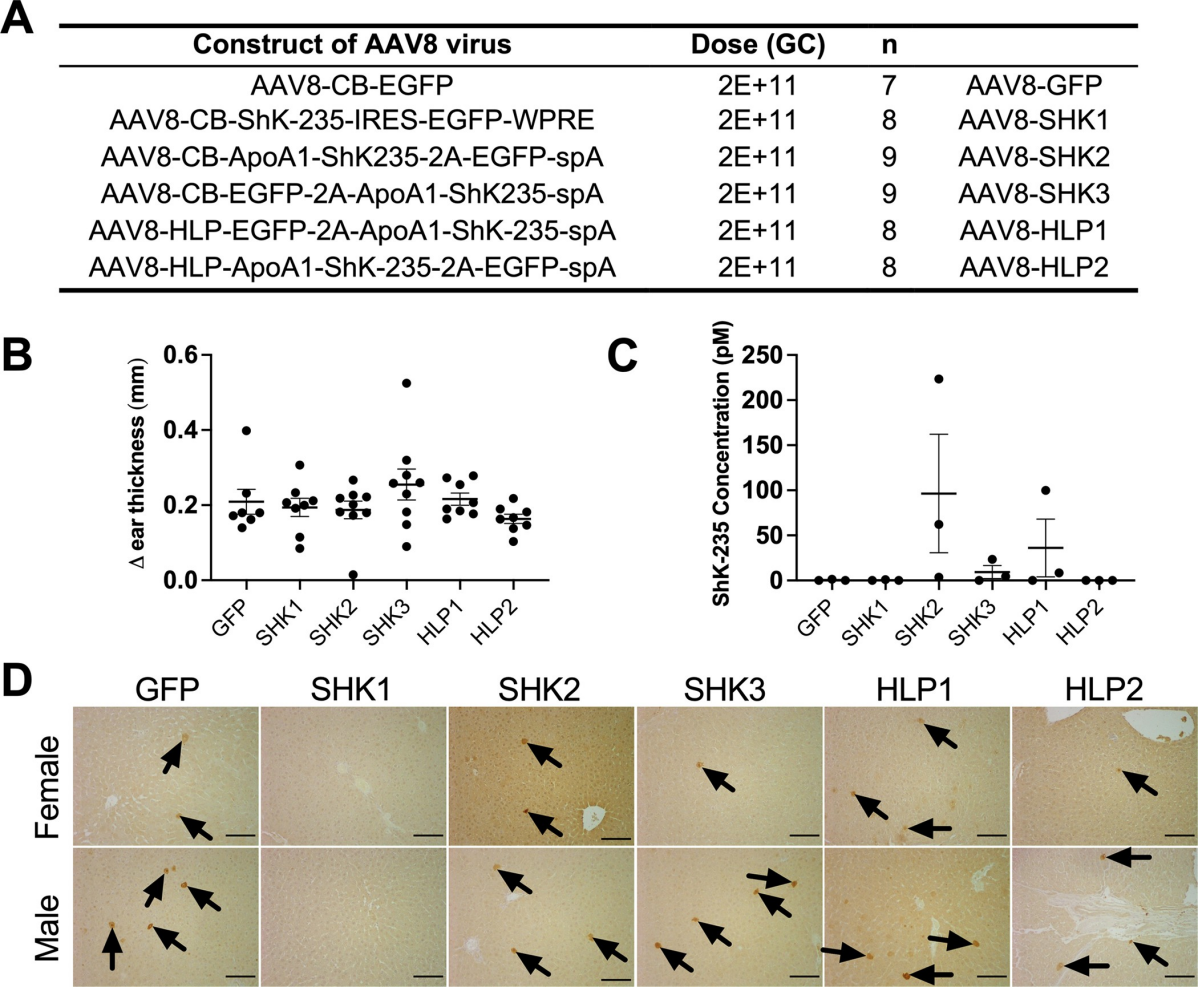
Lack of efficacy of the AAV8-based delivery of ShK-235 in Lewis rats. **(A)** AAV8 constructs injected intraperitoneally into rats, and their doses. N = 7–9 rats per group. **(B)** Difference (Δ) in ear thickness in rats immunized against ovalbumin and challenged with ovalbumin in one ear and with saline in the other. Each data point represents a different rat. Data from males and females are combined. **(C)** ShK-235 concentration in serum from rats injected with each of the different AAV8. Each serum sample was tested on 3 cells each. **(D)** Representative IHC images of livers from rats with DTH, showing GFP expression. The black arrows point to EGFP^+^ cells. Scale bars, 100 μm.

To test the ability of AAV8-ShK-235 to reduce T_EM_ lymphocyte-mediated inflammation, we had to switch our animal model to rats because rat T lymphocytes, but not mouse T cells, mimic human T lymphocytes in terms of Kv1.3 and KCa3.1 channel phenotype and function during inflammation. Similar to human T_EM_ cells, rat T_EM_ cells are exquisitely sensitive to Kv1.3 blockers. In contrast, mouse T cells express K^+^ channels other than just Kv1.3 and KCa3.1, do not upregulate Kv1.3 during differentiation into T_EM_ cells, and display little sensitivity to Kv1.3 blockers [24, 25, 56, 57]. Six groups of rats received one of the six vectors previously tested in mice (Fig 4A). No significant difference in reducing DTH could be observed in rats having received any of the ShK-235 expressing vectors compared to the AAV8-GFP control (Fig 4A and 4B). Patch-clamp results yielded no Kv1.3 blocking activity in the AAV8-GFP control, and only the SHK2 vector induced a low amount of ShK-235 in the circulation (96.3 ± 65.7 pM). The ShK-235 concentration of all other vectors was well below the IC_50_ of ShK-235 for Kv1.3 (Fig 4C). A low EGFP expression was detected in liver sections from all groups (Fig 4D).

### AAV8 inefficiently transduces Lewis rat hepatocytes

**Fig 5:**
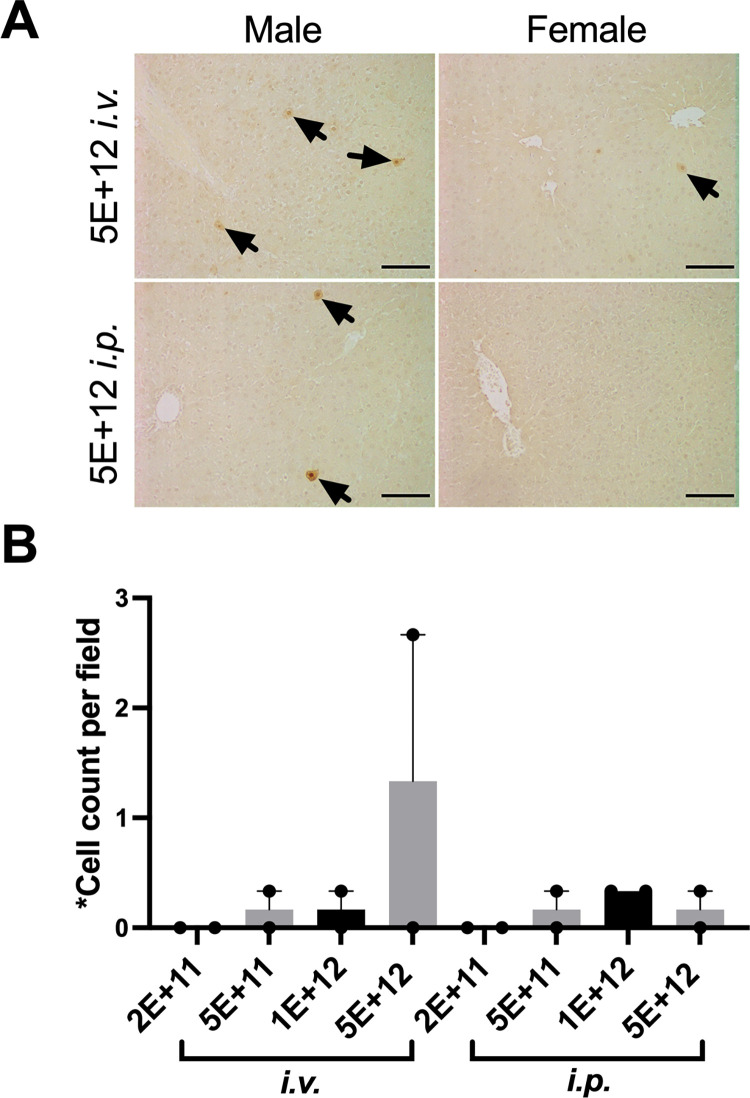
AAV8 has a low tropism for Lewis rat hepatocytes. **(A)** Representative IHC images showing EGFP expression in the livers of rat that received the highest dose (5E+12 GC) of AAV8-GFP intravenously (top) or intraperitoneally (bottom), The black arrows point to EGFP^+^ cells. Scale bars = 100 μm. **(B)** The number of EGFP positive hepatocytes per field area (13.2 mm^2^) in male and female rat livers. N = 2 per group. Mean ± SEM.

The AAV8-GFP vector was used to determine whether the number of AAV particles and their delivery routes impact rat hepatocyte transduction rates. Rats received a single intraperitoneal or intravenous injection of 2E+11 - 5E+12 GC of AAV8-GFP. Scaled for body size relative to mice, a dose of 1E+12 GC/rat should be sufficient to transduce >99% of hepatocytes. Only sparse EGFP-positive cells were detected in the liver of rats two weeks after injection of the highest dose of AAV8-GFP, and none in other groups, and no difference was seen between the injection route (Fig 5A and 5B). Despite consistent results from numerous studies with AAV8 liver transduction in mice, dogs, non-human primates, and human clinical trials [58–61], this serotype is remarkably inefficient in Lewis rats.

## Discussion

We show that plasmids encoding for the selective Kv1.3 blocking peptide ShK-235 are effective in expressing EGFP and in producing and secreting large amounts of functional ShK-235 by HEK293T cells. AAV8 vectors designed to induce the expression of EGFP and the secretion of ShK-235 by hepatocytes were very effective in mouse but not rat livers. AAV8 vectors designed to induce the production and secretion of ShK-235 by hepatocytes did not prevent weight gain in mice fed a HFD or a DTH reaction in rats, two conditions described in the literature to be altered by Kv1.3 blockers [19, 20, 24, 28].

When testing the constructs for efficiency of EGFP expression in HEK293T cells, we found that SHK3 produced the most EGFP and SHK1 the least, SHK2 being in between. The likely reason for this is the proximity of EGFP to the CB promoter in SHK3, whereas in SHK1 EGFP is positioned further away, located behind the IRES. For the secretion of functional ShK-235 however, the efficacy does not follow the same trend. Whereas cells transfected with the SHK1 and SHK3 constructs produce high levels of ShK-235, the cells transfected with SHK2 produce less. In SHK2, ShK-235 is just before the P2A ribosomal skipping peptide. After cleavage, P2A leaves its first 20 amino acids on the C-terminus of the protein before it, ShK-235 in the case of construct SHK2. Modification of the C-terminus of ShK can alter its potency against Kv1.3 [47] and, since ShK-235 was detected using a functional assay, we likely detect less Kv1.3 block from this construct due to the P2A amino acids affecting either the folding of the peptide or its binding to the Kv1.3 channel.

AAV8 vectors encoding for ShK-235 driven by a HLP with the leader sequence from the human ApoA1 protein have high efficiency in transducing hepatocytes of both male and female mice at the 2-week time point. These constructs did not have detectable off-tissue expression in other tissues. At later times, EGFP was still expressed by most hepatocytes of the males but at a lower rate by females. This discrepancy between sexes has previously been reported and may be due to the differences in hormone types and levels [62].

Many studies have examined the biodistribution of AAV8 in non-human primates and rodents, but few reports have addressed the difference in transduction efficiency in delivering immunomodulators such as ShK analogs between mouse and rat livers. We found a low tropism of AAV8 for Lewis rat hepatocytes. Since ShK and its analogs are not immunogenic [18, 27], decreased expression of the peptide is unlikely due to humoral immune response to the peptide itself. However, humoral immune responses to the AAV capsid can occur in rodent models [63]. We further confirmed that the low transduction efficiency is not affected by the route of delivery or by the number of viral particles administered. Our findings are in agreement with a previous publication using Gunn rats which Jurgen *et al*. compared AAV1, 2, 6 and 8, and found that vectors of the AAV1 serotype are more efficient for the transduction of Gunn rat hepatocytes in *vivo* but still less than for the transduction of mouse hepatocytes [64]. Furthermore, in Gunn rats, portal vein injection of AAV8 is more efficient, but the increase in efficacy is negligible when compared with the efficiency seen in mice [65]. Interestingly, we observed the “hot spot” effects reported in the literature [64, 66] in which a few rat hepatocytes express high levels of EGFP while most other rat hepatocytes do not. It is tempting to speculate that these transduced cells have a different set of cell-surface receptors that can bind AAV8 with high affinity relative to the remainder of the rat liver. It is also possible that the rats have pre-existing immunity and pre-formed antibodies that block AAV8 transduction, as seen in dogs, primates, and humans [67]. Given the importance of rat models of autoimmune diseases, further work is required to test the delivery efficiency and function of the AAV-ShK-235 *in vivo*. Such studies could utilize naturally occurring or genetically modified serotypes with an increased affinity for rat liver, such as AAV1 and adenoviruses, which have been demonstrated to efficiently transduce rat cells [65, 68].

Despite the concentration of functional ShK-235 being well above the IC_50_ for Kv1.3 block in the short-term, we did not replicate the previously described anti-obesity effects of Kv1.3 blockade or knock-down [19, 20]. Our results are similar to those obtained in rats treated with another Kv1.3 blocker, the scorpion toxin Vm24 [69]. One explanation could be that the concentration of circulating ShK-235 after AAV delivery may not be sufficient to inhibit weight gain, although this is unlikely because we detected circulating concentrations of ShK-235 as high as 18-fold the peptide’s IC_50_ on Kv1.3 [17], and no changes in weight gain were seen in mice injected with synthetic ShK-235. Second, Upadhyay et al. used ShK-186 rather than ShK-235, a different analog of the ShK toxin. Its single metabolite, the dephosphorylated form of ShK-186, called ShK-198 [30], is not selective for Kv1.3 over Kv1.1 compared with ShK-235 and Vm24 [17, 70] (for sequences of ShK, ShK-186, ShK-198, and ShK-235, see S8 Fig). In contrast, ShK-235 and Vm24 do not block Kv1.1 channels. It is possible that the anti-obesity effects observed in Upadhyay et al.’s work are due in part to Kv1.1 block. This would need to be tested with a selective Kv1.1 blocker [71]. A third possibility is that ShK-186, but not ShK-235 or Vm24, can access the olfactory bulb, as resistance to diet-induced obesity is olfactory bulb-dependent [72, 73].

Clinical deployment of immunomodulatory peptides could require more tunable control of expression than can be achieved with the current vector design. To our knowledge, overexpression of ShK-235 should have no deleterious effects on hepatocytes. The liver has an extraordinary secretory capacity, producing the vast majority of all secreted proteins in the circulation. It is unlikely that there could be cytotoxic effects resulting from saturation of the secretory machinery. Hepatocytes do not express the Kv1.3 channel targeted by ShK-235, so paracrine effects on these cells would also not be a major concern. Nonetheless, if there are unintended negative effects of ShK-235, an improved vector design could include chemical [74] or siRNA [75] switches to turn on or off expression of this peptide. This is an important future direction for the development of liver-derived AAV biologics, especially for autoimmune and inflammatory diseases.

In summary, we established an AAV8-based gene delivery system by which ShK-235 could be targeted to hepatocytes and conclude that AAV8-ShK-235 is a useful tool to deliver ShK-235 in mice, and a single-injection of AAV8-ShK-235 vectors can achieve short-term stable gene expression in mouse hepatocytes. Lastly, we report that AAV8 tropism for rat liver is surprisingly low, presenting technical challenges for testing in this animal model for autoimmune diseases. Our results demonstrate a limited potential of AAV8-based gene delivery of ShK-235 in transducing rodent hepatocytes for immunomodulation, alternative delivery methods should be sought out to facilitate wider clinical use of the immunomodulating peptide ShK-235.

## Supporting information

S1 Fig Vector diagrams. Inverted Terminal Repeat (ITR), Cytomegalovirus Enhancer Element (CMV Enh), Chicken beta Actin Promoter (CB), Simian Virus 40 intron (SV40 Intron), Enhanced Green Fluorescent Protein (EGFP), Woodchuck Hepatitis Virus Posttranscriptional Regulatory Element (WPRE), Bovine Growth Hormone Polyadenylation Signal (BGH polyA), human Apolipoprotein A-I signal peptide (hAPOA1-S.P.), Internal Ribosomal Entry Site (IRES), P2A Ribosomal Skipping Peptide (P2A), *Stichodactyla helianthus* derived peptide number 235 (ShK-235), small synthetic polyadenylation signal (sPA), Hybrid Liver Promoter (HLP).
(PDF)

S2 Fig Complete vector sequences. (PDF)

S3 Fig Sequences of human and mouse Apo A1. The sequences of human and mouse Apo A1 are aligned, showing amino acid homology (*). The signal peptide is indicated in blue and the pro-region in red. Both signal peptide and the pro-region of Apo A1 are included in our constructs highlighted in yellow).
(PDF)

S4 Fig Dose-response of synthetic ShK-235 on mKv1.3. A, Representative traces of the block of mKv1.3 currents by 100 pM synthetic ShK-235. B, Dose-response of mKv1.3 block by synthetic ShK-235. N = 3 cells per concentration. IC50 = 70.9 ± 21.9 pM.
(PDF)

S5 Fig EGFP expression in the heart, pancreas, kidney, lung, and adipose tissue of mice. Tissues from mice in Fig 2A stained for the expression of EGFP by immunohistochemistry. Black arrows point to stained cells. Scale bars = 100 μm.
(PDF)

S6 Fig Western blot of mice received AAV8-GFP or AAV8-HLP1 injections for 2 weeks. A-B, Western blot analysis of EGFP in liver lysates from mice injected with AAV8 constructs in Fig 2A with β-tubulin (β-Tub) used as a loading control. C-D, Western blot analysis of male and female mouse liver lysates from obesity mice received AAV8-GFP or AAV8-HLP1 injections, with β-tubulin used as a loading control.
(PDF)

S7 Fig Food intake of mice in the obesity trial. A, Food intake of the male mice. B, Food intake of the female mice. The food intake was calculated from the food added in each cage of 3 mice and the food remaining in each cage at the end of the week, before addition of new food. ShK-235 (■) 500 μg/kg daily, subcutaneously, n = 9 mice; Vehicle (●) 200 μl daily subcutaneously, n = 9 mice; AAV8-ShK-235 (▲) 2E+11 viral genome copies intraperitoneally n = 9 mice,; AAV8-GFP (▼) 2E+11 viral genome copies intraperitoneally, n = 9 mice. None of the data are statistically significantly different.
(PDF)

S8 Fig Amino acid sequences of ShK, its synthetic analog ShK-186, and its recombinant analog ShK-235. Differences between sequences are shown in bold and red. ShK is the 37 amino acid peptide originally isolated from the venom of the sea anemone *Stichodactyla helianthus* [22]. ShK-186 contains a pTyr attached to the peptide’s N-terminus via a 9-carbon atom linker (AEEA) that precludes its recombinant production. ShK-198 is the metabolite of ShK-186 and its N-terminal Tyr is dephosphorylated. ShK-235 differs from ShK by a Q16K substitution, an I21M substitution, and the addition of an Ala to the C-terminus.
(PDF)

S1 Raw images (PDF)
