# Supplementary material for: Adeno-Associated virus 8 delivers an immunomodulatory peptide to mouse liver more efficiently than to rat liver
Source: PLoS One. 2023 Apr 11;18(4):e0283996. doi: 10.1371/journal.pone.0283996 (PMC10089316; doi:10.1371/journal.pone.0283996)

**S1 Fig. Vector diagrams.**
Inverted Terminal Repeat (ITR), Cytomegalovirus Enhancer Element (CMV Enh), Chicken beta Actin Promoter (CB), Simian Virus 40 intron (SV40 Intron), Enhanced Green Fluorescent Protein (EGFP), Woodchuck Hepatitis Virus Posttranscriptional Regulatory Element (WPRE), Bovine Growth Hormone Polyadenylation Signal (BGH polyA), human Apolipoprotein A-I signal peptide (hAPOA1-S.P.), Internal Ribosomal Entry Site (IRES), P2A Ribosomal Skipping Peptide (P2A), *Stichodactyla helianthus* derived peptide number 235 (ShK-235), small synthetic polyadenylation signal (sPA), Hybrid Liver Promoter (HLP).

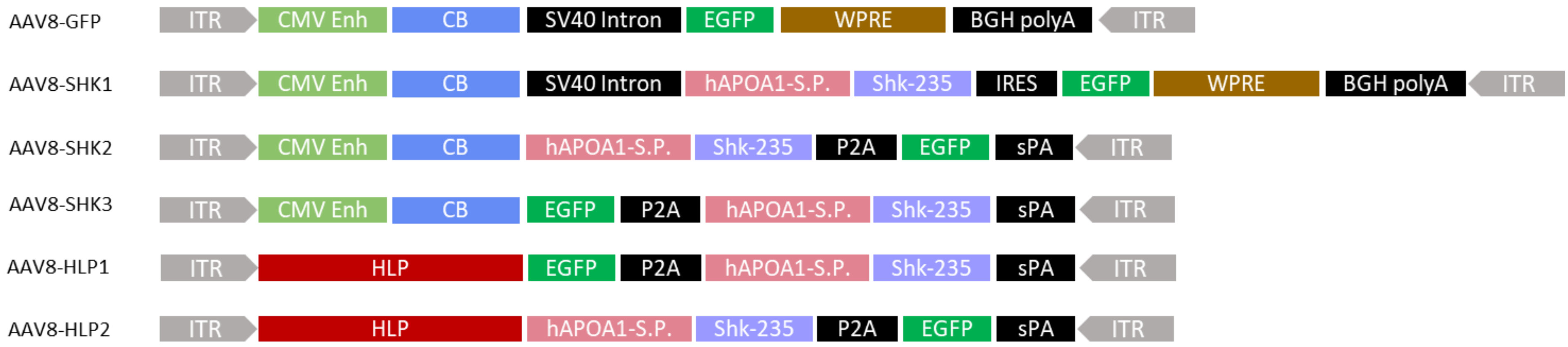

Supplement: S1 Fig — Inverted Terminal Repeat (ITR), Cytomegalovirus Enhancer Element (CMV Enh), Chicken beta Actin Promoter (CB), Simian Virus 40 intron (SV40 Intron), Enhanced Green Fluorescent Protein (EGFP), Woodchuck Hepatitis Virus Posttranscriptional Regulatory Element (WPRE), Bovine Growth Hormone Polyadenylation Signal (BGH polyA), human Apolipoprotein A-I signal peptide (hAPOA1-S.P.), Internal Ribosomal Entry Site (IRES), P2A Ribosomal Skipping Peptide (P2A), Stichodactyla helianthus derived peptide number 235 (ShK-235), small synthetic polyadenylation signal (sPA), Hybrid Liver Promoter (HLP). (PDF) [file pone.0283996.s001.pdf]
