# Supplementary material for: Adeno-Associated virus 8 delivers an immunomodulatory peptide to mouse liver more efficiently than to rat liver
Source: PLoS One. 2023 Apr 11;18(4):e0283996. doi: 10.1371/journal.pone.0283996 (PMC10089316; doi:10.1371/journal.pone.0283996)

S3 Fig. Sequences of human and mouse Apo A1. The sequences of human and mouse Apo A1 are aligned, showing amino acid homology (*). The signal peptide is indicated in blue and the pro-region in red. Both signal peptide and the pro-region of Apo A1 are included in our constructs highlighted in yellow).

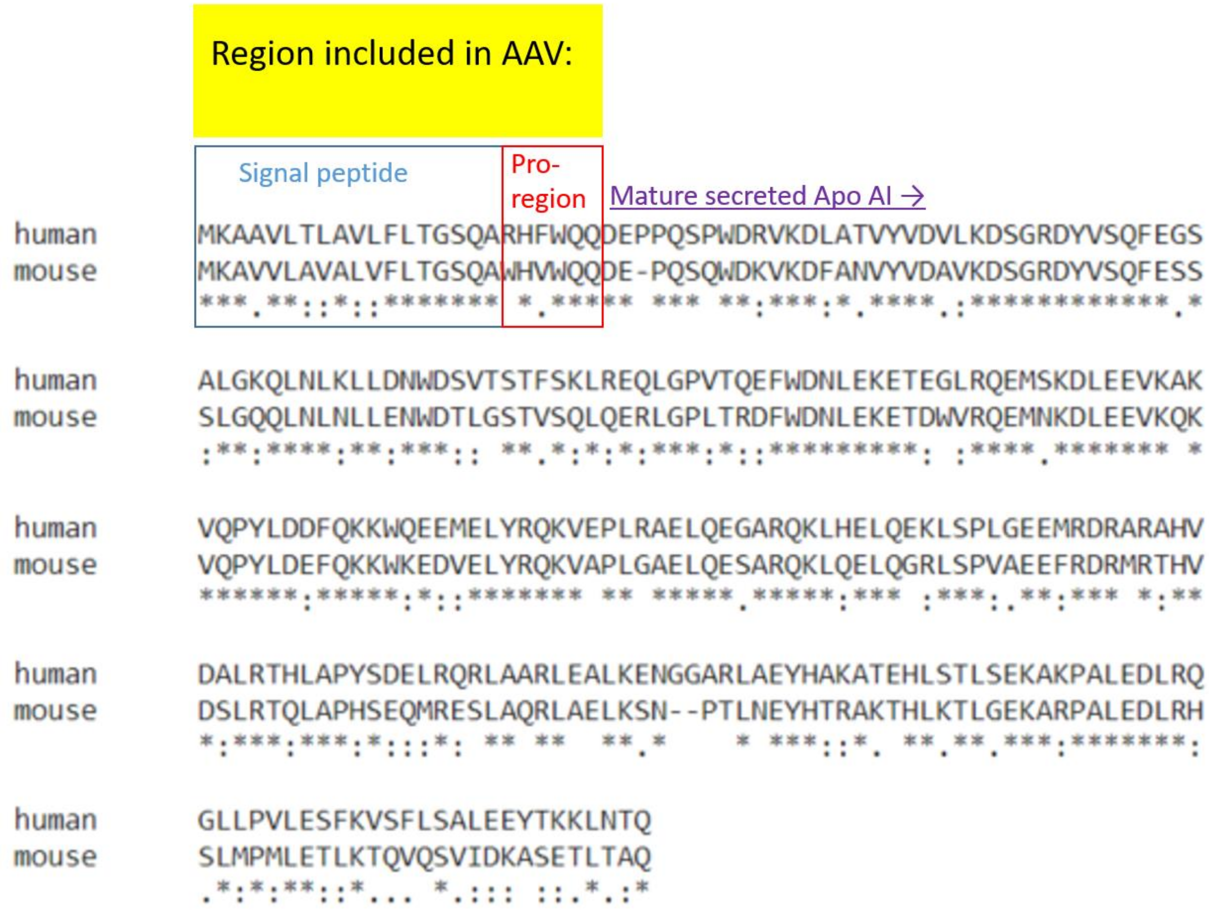

Supplement: S3 Fig — The sequences of human and mouse Apo A1 are aligned, showing amino acid homology (*). The signal peptide is indicated in blue and the pro-region in red. Both signal peptide and the pro-region of Apo A1 are included in our constructs highlighted in yellow). (PDF) [file pone.0283996.s003.pdf]
