# Supplementary material for: Adeno-Associated virus 8 delivers an immunomodulatory peptide to mouse liver more efficiently than to rat liver
Source: PLoS One. 2023 Apr 11;18(4):e0283996. doi: 10.1371/journal.pone.0283996 (PMC10089316; doi:10.1371/journal.pone.0283996)

S4 Fig. Dose-response of synthetic ShK-235 on mKv1.3. A, Representative traces of the block of mKv1.3 currents by 100 pM synthetic ShK-235. B, Dose-response of mKv1.3 block by synthetic ShK-235. N = 3 cells per concentration. IC_{50} =70.9 ± 21.9 pM.

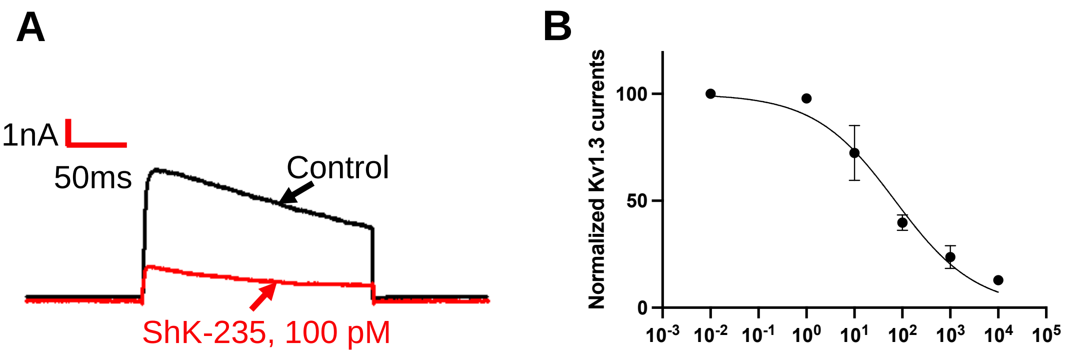

Supplement: S4 Fig — A, Representative traces of the block of mKv1.3 currents by 100 pM synthetic ShK-235. B, Dose-response of mKv1.3 block by synthetic ShK-235. N = 3 cells per concentration. IC50 = 70.9 ± 21.9 pM. (PDF) [file pone.0283996.s004.pdf]
