# Supplementary material for: Adeno-Associated virus 8 delivers an immunomodulatory peptide to mouse liver more efficiently than to rat liver
Source: PLoS One. 2023 Apr 11;18(4):e0283996. doi: 10.1371/journal.pone.0283996 (PMC10089316; doi:10.1371/journal.pone.0283996)

S5 Fig. EGFP expression in the heart, pancreas, kidney, lung, and adipose tissue of mice. Tissues from mice in Fig. 2A stained for the expression of EGFP by immunohistochemistry. Black arrows point to stained cells. Scale bars = 100 µm.

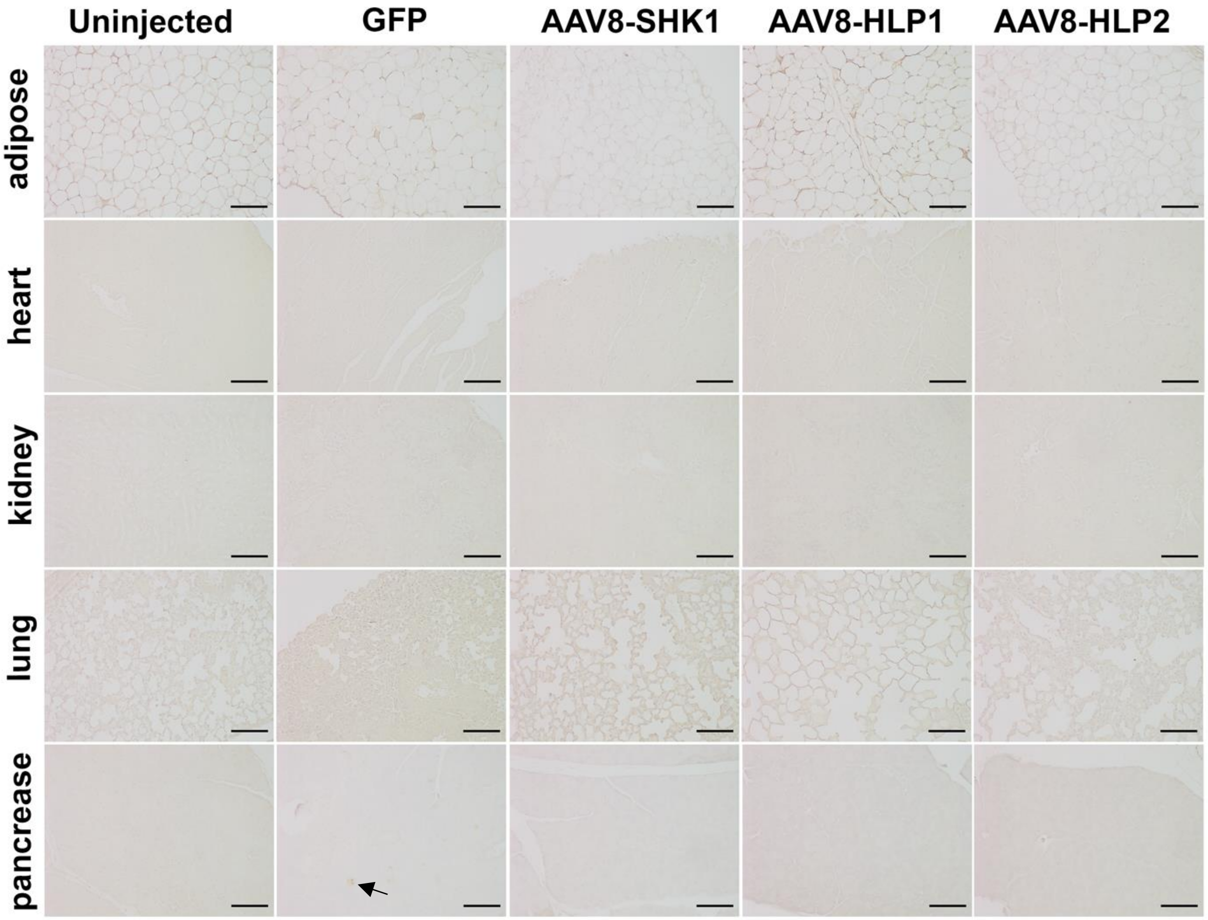

Supplement: S5 Fig — Tissues from mice in Fig 2A stained for the expression of EGFP by immunohistochemistry. Black arrows point to stained cells. Scale bars = 100 μm. (PDF) [file pone.0283996.s005.pdf]
