# Supplementary material for: Adeno-Associated virus 8 delivers an immunomodulatory peptide to mouse liver more efficiently than to rat liver
Source: PLoS One. 2023 Apr 11;18(4):e0283996. doi: 10.1371/journal.pone.0283996 (PMC10089316; doi:10.1371/journal.pone.0283996)

S6 Fig. Western blot of mice received AAV8-GFP or AAV8-HLP1 injections for 2 weeks. A-B, Western blot analysis of EGFP in liver lysates from mice injected with AAV8 constructs in Fig. 2A with β-tubulin (β-Tub) used as a loading control. C-D, Western blot analysis of male and female mouse liver lysates from obesity mice received AAV8-GFP or AAV8-HLP1 injections, with β-tubulin used as a loading control.

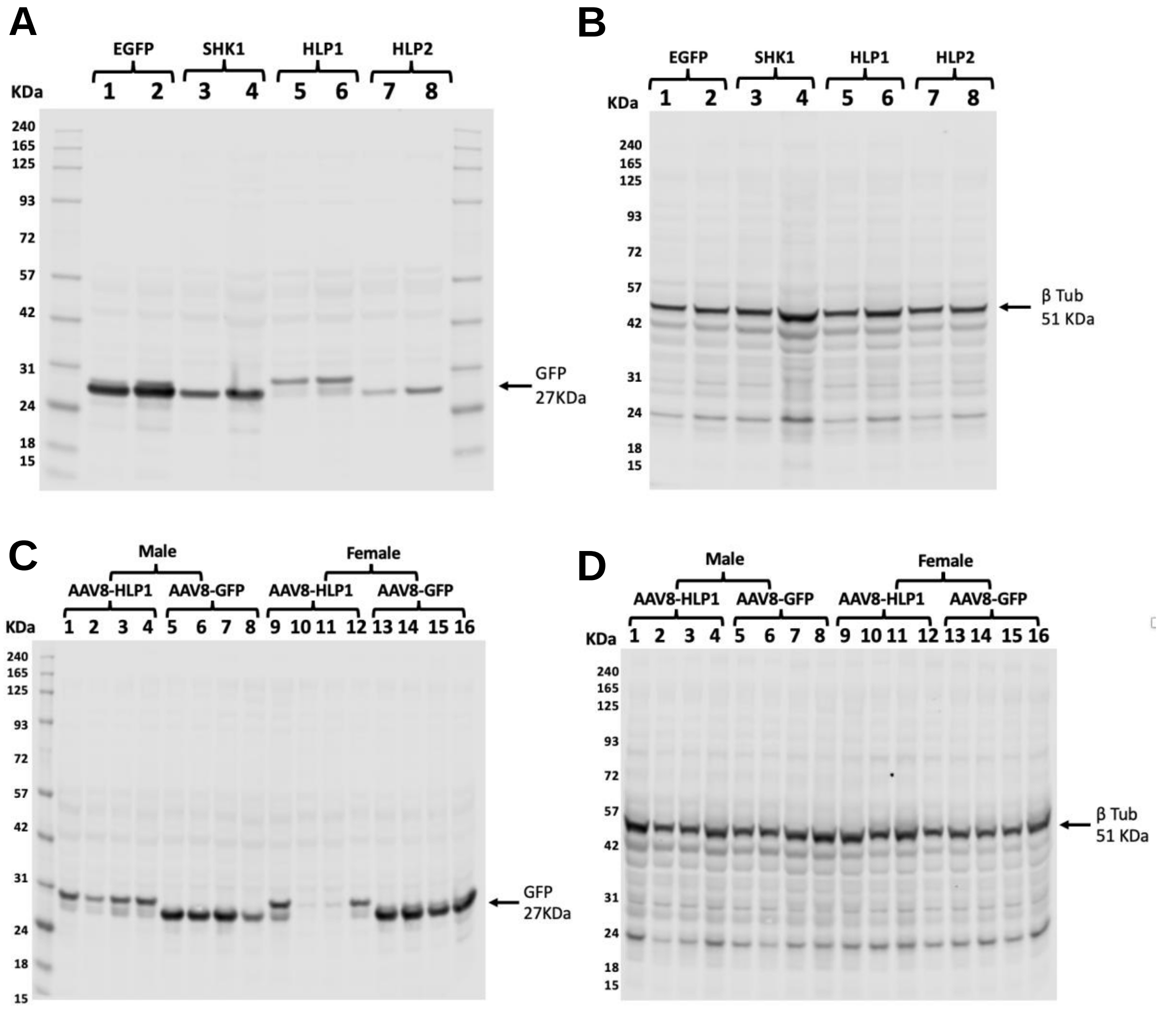

Supplement: S6 Fig — A-B, Western blot analysis of EGFP in liver lysates from mice injected with AAV8 constructs in Fig 2A with β-tubulin (β-Tub) used as a loading control. C-D, Western blot analysis of male and female mouse liver lysates from obesity mice received AAV8-GFP or AAV8-HLP1 injections, with β-tubulin used as a loading control. (PDF) [file pone.0283996.s006.pdf]
