# Supplementary material for: Adeno-Associated virus 8 delivers an immunomodulatory peptide to mouse liver more efficiently than to rat liver
Source: PLoS One. 2023 Apr 11;18(4):e0283996. doi: 10.1371/journal.pone.0283996 (PMC10089316; doi:10.1371/journal.pone.0283996)

S7 Fig. Food intake of mice in the obesity trial.

A, Food intake of the male mice. B, Food intake of the female mice. The food intake was calculated from the food added in each cage of 3 mice and the food remaining in each cage at the end of the week, before addition of new food. ShK-235 (■) 500 µg/kg daily, subcutaneously, n = 9 mice; Vehicle (●) 200 µl daily subcutaneously, n = 9 mice; AAV8-ShK-235 (▲) 2E+11 viral genome copies intraperitoneally n = 9 mice,; AAV8-GFP (▼) 2E+11 viral genome copies intraperitoneally, n = 9 mice. None of the data are statistically significantly different.

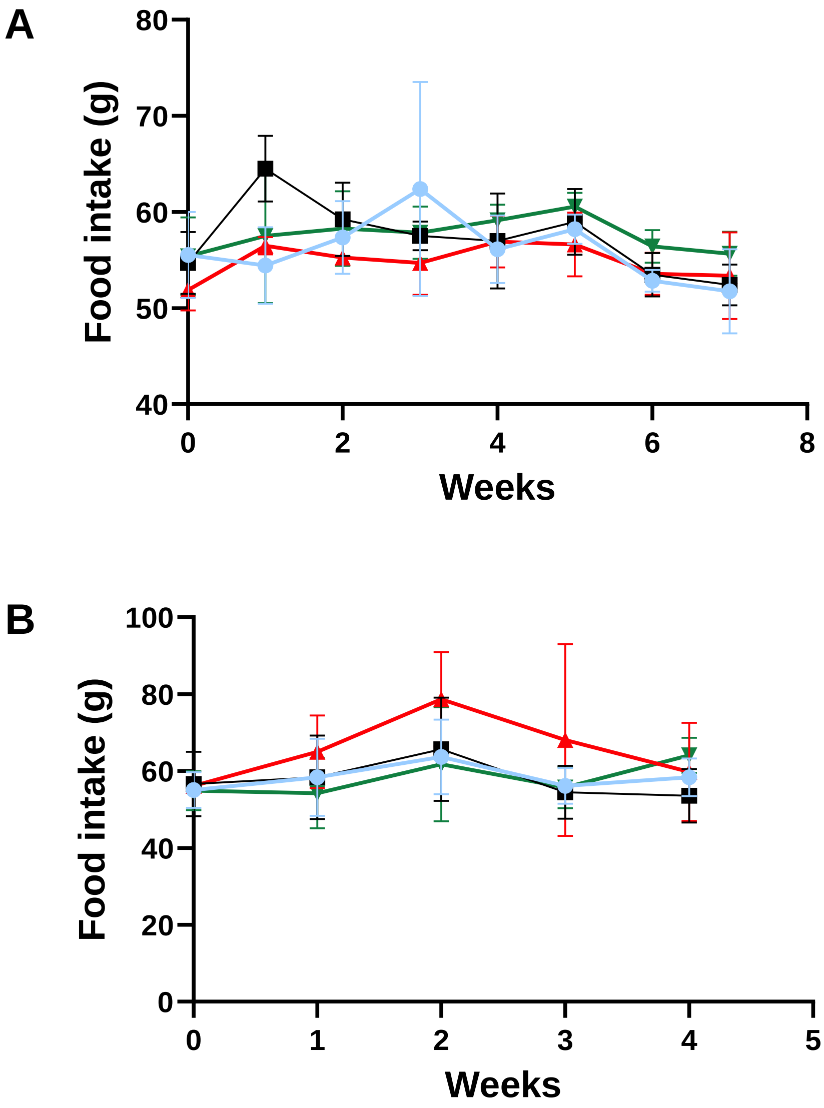

Supplement: S7 Fig — A, Food intake of the male mice. B, Food intake of the female mice. The food intake was calculated from the food added in each cage of 3 mice and the food remaining in each cage at the end of the week, before addition of new food. ShK-235 (■) 500 μg/kg daily, subcutaneously, n = 9 mice; Vehicle (●) 200 μl daily subcutaneously, n = 9 mice; AAV8-ShK-235 (▲) 2E+11 viral genome copies intraperitoneally n = 9 mice,; AAV8-GFP (▼) 2E+11 viral genome copies intraperitoneally, n = 9 mice. None of the data are statistically significantly different. (PDF) [file pone.0283996.s007.pdf]
