# Supplementary material for: Adeno-Associated virus 8 delivers an immunomodulatory peptide to mouse liver more efficiently than to rat liver
Source: PLoS One. 2023 Apr 11;18(4):e0283996. doi: 10.1371/journal.pone.0283996 (PMC10089316; doi:10.1371/journal.pone.0283996)
Supplement: S8 Fig — Differences between sequences are shown in bold and red. ShK is the 37 amino acid peptide originally isolated from the venom of the sea anemone Stichodactyla helianthus [22]. ShK-186 contains a pTyr attached to the peptide’s N-terminus via a 9-carbon atom linker (AEEA) that precludes its recombinant production. ShK-198 is the metabolite of ShK-186 and its N-terminal Tyr is dephosphorylated. ShK-235 differs from ShK by a Q16K substitution, an I21M substitution, and the addition of an Ala to the C-terminus. (PDF) [file pone.0283996.s008.pdf]

S8 Fig. Amino acid sequences of ShK, its synthetic analog ShK-186, and its recombinant analog ShK-235.

Differences between sequences are shown in bold and red. ShK is the 37 amino acid peptide originally isolated from the venom of the sea anemone *Stichodactyla helianthus* (1). ShK-186 contains a pTyr attached to the peptide's N-terminus via a 9-carbon atom linker (AEEA) that precludes its recombinant production. ShK-198 is the metabolite of ShK-186 and its N-terminal Tyr is dephosphorylated. ShK-235 differs from ShK by a Q16K substitution, an I21M substitution, and the addition of an Ala to the C-terminus.

ShK RSCIDTIPKSRCTAFQCKHSMKYRLSFCRKTCGTC
ShK-186 **pTyr-AEEA-**RSCIDTIPKSRCTAFQCKHSMKYRLSFCRKTCGTC
ShK-198 **Tyr-AEEA-**RSCIDTIPKSRCTAFQCKHSMKYRLSFCRKTCGTC
ShK-235 RSCIDTIPKSRCTAF**K**CKHS**I**KYRLSFCRKTCGTC**A**

1. O. Castañeda *et al.*, Characterization of a potassium channel toxin from the Caribbean Sea anemone Stichodactyla helianthus. *Toxicon* **33**, 603-613 (1995).
