# Supplementary material for: Adeno-Associated virus 8 delivers an immunomodulatory peptide to mouse liver more efficiently than to rat liver
Source: PLoS One. 2023 Apr 11;18(4):e0283996. doi: 10.1371/journal.pone.0283996 (PMC10089316; doi:10.1371/journal.pone.0283996)

Supporting information

Adeno-Associated Virus 8 delivers an immunomodulatory peptide to mouse liver more efficiently than to rat liver

Yuqing Wang[1], Ayrea E. Hurley[1], Marco De Giorgi[1], Mark. R. Tanner[1], Rong-Chi Hu[1], Michael W. Pennington[2], William R. Lagor[1], Christine Beeton[1]

[1] Department of Integrative Physiology, Baylor College of Medicine, Houston, Texas, United State of America

[2] Ambiopharm, Inc., North Augusta, South Carolina, United State of America

S1 Fig. Western blot of mice received AAV8-GFP or AAV8-HLP1 injections for 2 weeks. **A-B**, Western blot analysis of EGFP in liver lysates from mice injected with AAV8 constructs in Fig. 2A with β-tubulin (β-Tub) used as a loading control. Figure 2C was generated from **S1Fig A-B**. **C-D**, Western blot analysis of male and female mouse liver lysates from obesity mice received AAV8-GFP or AAV8-HLP1 injections, with β-tubulin used as a loading control. Figure 3D was generated from **S1Fig C-D**. Visualization was performed with Odyssey and Image Studio, Intensity 5, 169 μm resolution.

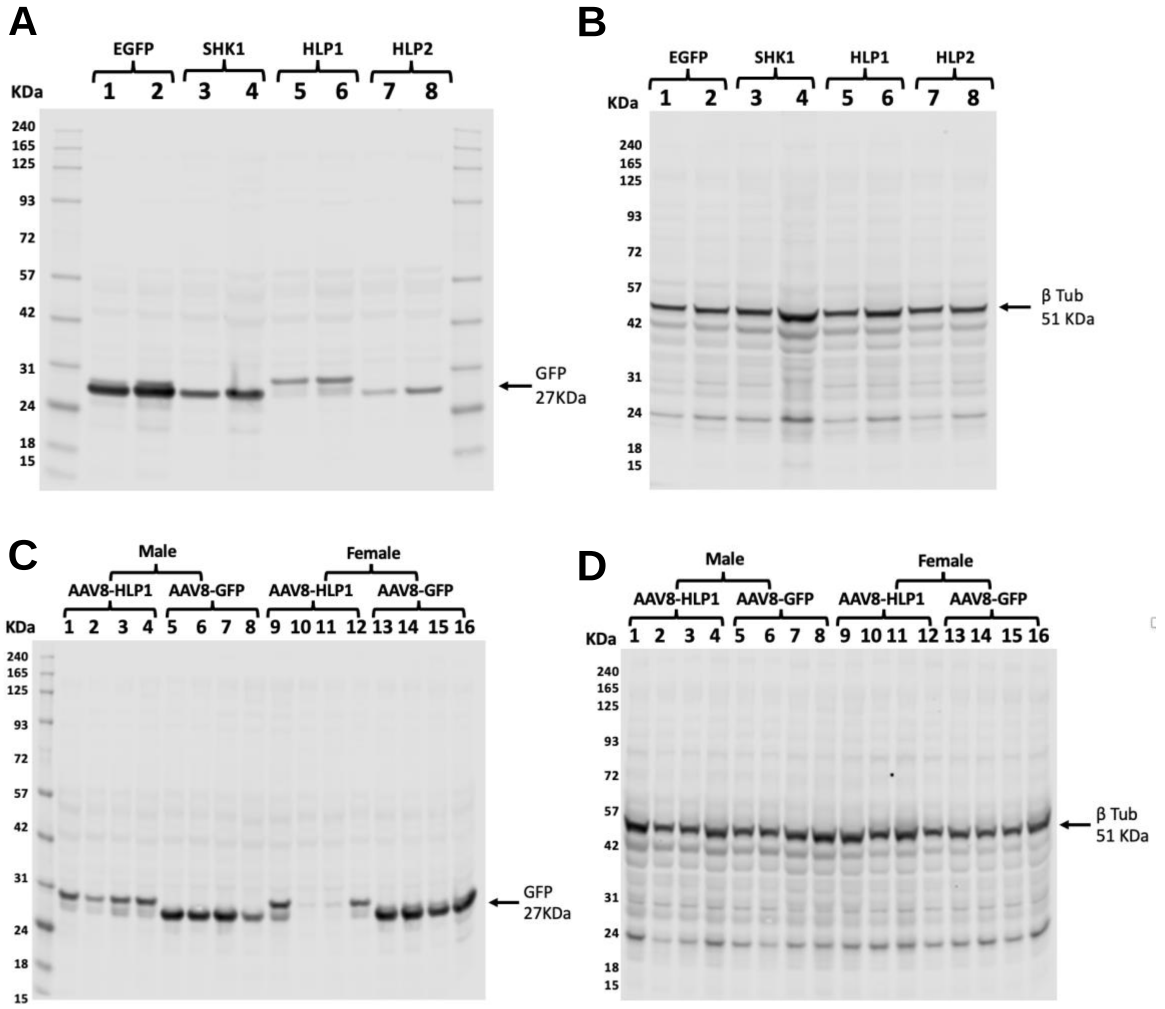

Supplement: S1 Raw images — (PDF) [file pone.0283996.s009.pdf]
